# Role of KNDy Neurons Expressing Kisspeptin, Neurokinin B, and Dynorphin A as a GnRH Pulse Generator Controlling Mammalian Reproduction

**DOI:** 10.3389/fendo.2021.724632

**Published:** 2021-09-09

**Authors:** Yoshihisa Uenoyama, Mayuko Nagae, Hitomi Tsuchida, Naoko Inoue, Hiroko Tsukamura

**Affiliations:** Laboratory of Animal Reproduction, Graduate School of Bioagricultural Sciences, Nagoya University, Nagoya, Japan

**Keywords:** *Kiss1*, *Tac3*, *Pdyn*, GPR54, NK3 receptor, κ-opioid receptor

## Abstract

Increasing evidence accumulated during the past two decades has demonstrated that the then-novel kisspeptin, which was discovered in 2001, the known neuropeptides neurokinin B and dynorphin A, which were discovered in 1983 and 1979, respectively, and their G-protein-coupled receptors, serve as key molecules that control reproduction in mammals. The present review provides a brief historical background and a summary of our recent understanding of the roles of hypothalamic neurons expressing kisspeptin, neurokinin B, and dynorphin A, referred to as KNDy neurons, in the central mechanism underlying gonadotropin-releasing hormone (GnRH) pulse generation and subsequent tonic gonadotropin release that controls mammalian reproduction.

## Introduction

Peptides play critical roles in the nervous systems of both invertebrates and vertebrates alike. The release of peptides as intercellular signaling molecules is an evolutionarily ancient property of neurons (1, 2). In fact, the nervous systems in cnidarians, a class of mostly aquatic animals that have existed for over 630 million years, are mainly controlled by peptidergic signals (2). Several signaling peptides were discovered in cnidarians and most of them were characterized by C-terminal amidation (3). These so-called neuropeptides are stored in intracellular neurosecretory vesicles until being secreted by exocytosis. Once secreted, these neuropeptides act on target cells by binding to and activating plasma membrane receptors, leading to changes in the intracellular signaling system.

The G-protein-coupled receptors (GPCRs) represent the single largest family of plasma membrane receptors, encompassing about 860 members in humans (4). The concept that these GPCRs may form a supergene family was derived from the discovery in 1986 that the β2-adrenergic receptors and the opsins share a seven-transmembrane domain topology (5). At that time, the β2-adrenergic receptors and the opsins were not known to have much in common except for coupling to G-proteins to elicit the intracellular signaling system. The structural feature of the seven-transmembrane domain topology allowed a search for putative GPCRs in the genome, resulting in the discovery of a large number of GPCRs. The GPCRs are split into two major groups: olfactory and non-olfactory receptors. The non-olfactory GPCRs are further classified based on whether the endogenous ligand is known or unknown. Approximately 30% of the ~400 non‐olfactory human GPCRs have not been definitively paired with endogenous ligands and are designated as orphan GPCRs (6).

The present review focuses on neuropeptides, kisspeptin, neurokinin B and dynorphin A, and their GPCRs, because evidence accumulated during the past two decades has demonstrated that those three neuropeptides serve as key molecules that control reproduction *via* controlling pulsatile gonadotropin-releasing hormone (GnRH) release in mammals. More specifically, the hypothalamic arcuate nucleus (ARC) neurons co-expressing kisspeptin/neurokinin B/dynorphin A, referred to as “KNDy neurons”, are evident in mammals including rodents (7–11), ruminants (12–14), and primates (15, 16) and the KNDy neurons are now considered to be largely responsible for GnRH pulse generation. In this review, we provide a historical background on the concept of GnRH pulse generator to control gametogenesis and steroidogenesis in mammals and a summary of our recent understanding of the roles of KNDy neurons in the central mechanism underlying GnRH pulse generation and subsequent tonic release of gonadotropins.

## The Discovery of GnRH/Gonadotropin Pulses

One hundred years ago, in the 1920s, Smith (17) and Evans and Long (18) investigated the effects of hypophysectomy or the administration of pituitary extract on gonadal activities, and accordingly suggested that gonadal activities are regulated by one or more factors from the anterior pituitary gland (19). In the 1930s, luteinizing hormone (LH) and follicle-stimulating hormone (FSH) were successfully extracted from the anterior pituitary gland (20). The presence of GnRH [first named LH-RH/FSH-RH or LHRH (21)], which controls gonadotropin release *via* the pituitary portal circulation, was predicted by Harris and Jacobsohn because they showed that the structure and function of the transplanted pituitary gland were maintained only when the pituitary gland was relocated under the median eminence in rats (22). Consequently, GnRH, which stimulates both LH and FSH release, was isolated in the early 1970s by two independent laboratories headed by Schally and Guillemin, respectively (23, 24).

In the early 1970s, Knobil and colleagues first demonstrated tonic (pulsatile) and cyclic (surge) gonadotropin release in female rhesus monkeys, used as a model of humans (25, 26), and predicted that the pulsatile nature of tonic gonadotropin release is likely caused by pulsatile GnRH stimulation to the anterior pituitary gland. Their pioneer study demonstrated the indispensable role of GnRH pulses in the control of gonadotropin release from the anterior pituitary gland (27). Indeed, plasma LH and FSH levels were kept at physiological levels only when GnRH was administrated in a pulsatile manner at a physiological frequency (once per hour) in female rhesus monkeys with a hypothalamic lesion (which resulted in the lack of endogenous GnRH release). In contrast, continuous GnRH administration paradoxically inhibited gonadotropin release in these monkeys (27). These findings indicated that GnRH pulses are needed to sustain the normal response of the anterior pituitary gland to GnRH stimulation. This knowledge has been exploited towards human reproductive technology and therapies. Indeed, some patients take pulsatile GnRH administration by an attached pump as a medical treatment to enhance folliculogenesis and recover ovulation (28), whereas chronic GnRH treatment is therapeutically used to inhibit sex steroid release in patients suffering from endometriosis as well as prostate cancer (29).

The pulsatile GnRH release was first described in ewes by measurements of GnRH in the pituitary portal blood in 1982 (30) and then examined in more detail in 1992 (31). These studies demonstrated that GnRH pulses detected in the pituitary portal circulation synchronized with LH pulses detected in the peripheral circulation.

## Localization of the GnRH Pulse Generator

Pulsatile GnRH release from GnRH neurons into the pituitary portal circulation has been hypothesized to be driven by the mechanism of the so-called “GnRH pulse generator” (32, 33). The first experimental evidence for the localization of the GnRH pulse generator was provided by Halasz and Pupp (34): They designed a small knife cut to isolate the mediobasal hypothalamus (MBH), leaving the region in contact with the pituitary gland but devoid of neural connections with the other brain regions. This complete deafferentation of the MBH failed to affect the testicular function (spermatogenesis) in males nor ovarian function (folliculogenesis) except for ovulation in female rats. These findings indicated that the complete deafferentation of the MBH disrupts the preovulatory GnRH/LH surge but not the GnRH pulses that drive tonic gonadotropin release in female rats. Subsequent studies had confirmed that pulsatile LH release is not impaired by the complete hypothalamic deafferentation in rats (35, 36). Taken together, these findings indicated that the GnRH pulse generator would be located in the MBH. Importantly, GnRH neuronal cell bodies are mainly located in the preoptic area (POA) and GnRH nerve terminals are located in the median eminence within the MBH in rats, indicating that the GnRH neurons themselves may not be involved in GnRH pulse generation and GnRH terminals would be an effector of GnRH pulse generator (36, 37).

Knobil and colleagues then established an important method to monitor GnRH pulse generator activity in the MBH *via* an electrophysiological approach in rhesus monkeys (38): This approach revealed that rhythmic increases in the multiple unit activity (termed MUA volleys) were accompanied by LH pulses detected in the peripheral circulation, when the recording electrodes were placed within the MBH. The MUA is the summation of the electrical activity of multiple neurons around the electrodes, and the origin of MUA volleys remained unknown at that time. This method was subsequently adapted to rats and goats (39–41) and successfully showed the MUA volleys in the MBH accompanied by LH pulses in such species as well. From then, significant effort was made to identify the intrinsic sources of the GnRH pulse generator for many years.

## The ARC Kisspeptin Neurons Are a Major Regulator of GnRH/Gonadotropin Pulses

**Figure 1:**
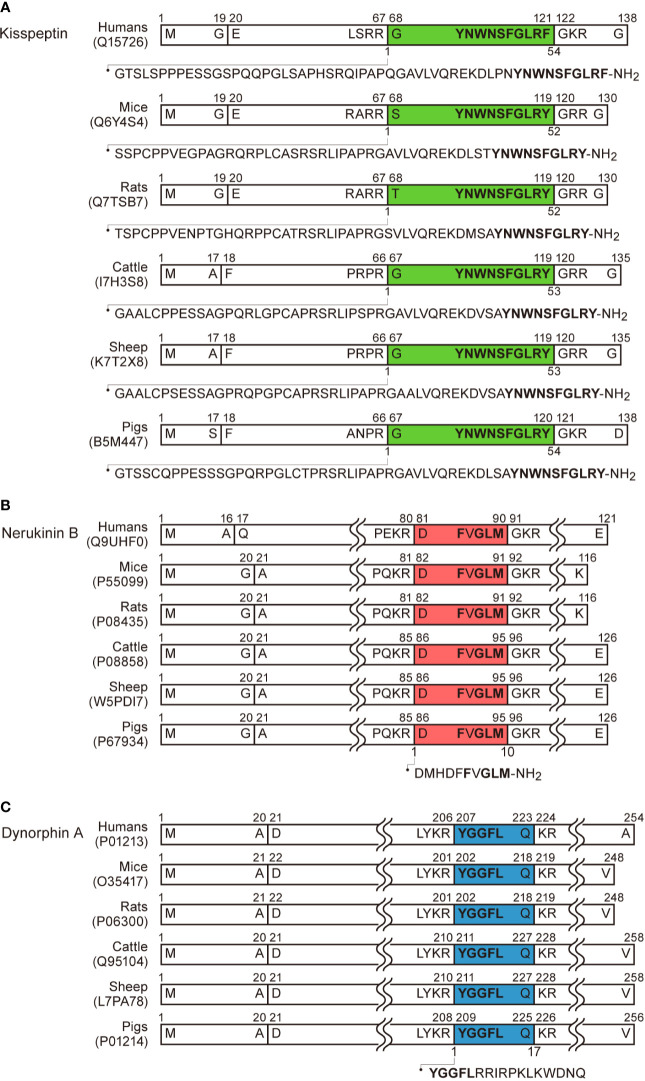
Schematic illustration of kisspeptin, neurokinin B, dynorphin A, and their precursors in humans, mice, rats, cattle, sheep, and pigs based on the previous reports (43, 45, 47, 49) and UniProtKB (https://www.uniprot.org/uniprot/). The precursors comprise a signal peptide in the N-terminal. **(A)** Kisspeptin consists of 52 or 54 amino acids cleaved from the precursors and the C-terminal is amidated. The C-terminal 10-amino acids (Kp-10) are identical among mice, rats, cattle, sheep, and pigs. Note that C-terminal tyrosine is replaced with phenylalanine in humans. **(B)** Neurokinin B consists of 10 amino acids cleaved from the precursors and the C-terminal is amidated. The amino acid sequence of neurokinin B is identical among humans, mice, rats, cattle, sheep, and pigs. The C-terminal amino acid sequence Phe-X-Gly-Leu-Met (or Leu)-amide is commonly found in tachykinin family peptides. **(C)** Dynorphin A consists of 17 amino acids cleaved from their precursors. The amino acid sequence of dynorphin A is identical among mice, rats, cattle, sheep, and pigs. The N-terminal amino acid sequence Tyr-Gly-Gly-Phe-Leu (or Met) are commonly found in endogenous opioid peptides.

At the turn of the twenty-first century, the discovery of kisspeptin provided a breakthrough in our understanding of the source of the GnRH pulse generator. Kisspeptin was first found as an endogenous ligand of GPR54, a then-orphan GPCR that shares significant homology with galanin receptors (42), from human placenta extract (43, 44). Kisspeptin was identified as a 54-amino-acid peptide cleaved from a 145-amino-acid prepropeptide in humans (43, 44). The C-terminal amidated 10-amino-acid sequence of the peptide (Kp-10), which is essential and sufficient for interaction with GPR54, a Gq-coupled stimulatory GPCR (43), is identical among mammals, except for the C-terminal phenylalanine which is changed to tyrosine in non-primate mammals (43–50). The precursor and mature kisspeptin with the C-terminal amidation in humans, rodents, and domestic animals are summarized in **Figure 1A**. Moreover, an indispensable role of kisspeptin as a neuropeptide in the central nervous system regulating reproduction in humans, was uncovered by two studies published in 2003 (51, 52): Two groups from the US and France independently demonstrated inactivating mutations of the *GPR54* gene in patients suffering from hypogonadotropic hypogonadism with pubertal failure. To date, several *Kiss1* or *Gpr54* knockout rodents replicated hypogonadotropic hypogonadism as seen in humans (52–58). Later, a Turkish group demonstrated that patients carrying inactivating mutations of the *KISS1* gene also exhibited hypogonadotropic hypogonadism (59). Taken together, these findings suggest that kisspeptin-GPR54 signaling serves as a key regulator for puberty onset and gonadotropin release in mammals.

Kisspeptin profoundly stimulated GnRH/gonadotropin release in mammals (53, 54, 60–63). The previous studies demonstrated that central administration of full-length kisspeptin or Kp-10 stimulated gonadotropin release in rodents (54, 60–62), ruminants (53), and primates (63). In addition, the stimulatory effect of kisspeptin on gonadotropin release was blocked by GnRH antagonists in both rodents (60, 61, 64) and primates (63), indicating that kisspeptin-induced gonadotropin release is mediated by GnRH. The previous *in vitro* study showed that kisspeptin stimulates GnRH release from the rat hypothalamic tissue *via* stimulatory Gq-protein-mediated activation of phospholipase C and mobilization of intracellular Ca^2+^ (65). Further, several histological analyses showed that *Gpr54* is expressed in a majority of GnRH neurons in mice (53, 66, 67) and rats (60, 68). Electrophysiological studies revealed that kisspeptin exerted a potent direct depolarizing effect on GnRH neurons (67, 69, 70). Further, GnRH neuron-specific *Gpr54* knockout mice resulted in infertility, whereas the rescuing *Gpr54* in GnRH neurons in global *Gpr54* knockout mice restored fertility (71), suggesting that GPR54 solely in GnRH neurons is enough for fertility in mice. Taken together, these findings suggest that kisspeptin directly stimulates GnRH release *via* GPR54 expressed in GnRH neurons to lead consequent gonadotropin secretion in mammals.

There are two major populations of hypothalamic kisspeptin neurons: one population is localized in the ARC—inside the MBH—in most mammals examined to date, and the other population is localized more rostral regions such as the anteroventral periventricular nucleus (AVPV) in rodents (72–77) and the POA in most of the other mammals including ruminants (12, 14, 46, 78–83), primates (50, 63, 84, 85), and others (47, 48). The role of AVPV/POA kisspeptin neurons in generating GnRH/LH surge in females was reviewed elsewhere (86–88). Circumstantial evidence suggesting that ARC kisspeptin neurons are an intrinsic source of the GnRH pulse generator has been accumulated as follows. When the MUA is measured in goats through recoding electrodes targeted to the vicinity of the ARC kisspeptin neurons, MUA volleys are found at regular intervals and are synchronized with LH pulses (13, 46). Further, recent *in vivo* GCaMP6 fiber photometry technology revealed that ARC kisspeptin neurons exhibited rhythmic increases in intracellular Ca^2+^ accompanied by LH pulses in mice (89, 90). Optogenetic stimulation of ARC kisspeptin neurons induced and optogenetic inhibition of ARC kisspeptin neurons suppressed LH pulses in kisspeptin neuron-specific channelrhodopsin- and archaerhodopsin-expressed gene-modified mice, respectively (89, 91).

GnRH neuronal axons in the median eminence seem to be an action site of kisspeptin for the generation of GnRH pulses. Immunoelectron microscopy revealed that kisspeptin and GnRH fibers are closely associated with each other in the internal layer of the median eminence and that few typical synaptic structures were found between kisspeptin and GnRH fibers in rats and goats, suggesting that kisspeptin acts on GnRH axons in a non-synaptic manner, such as “volume transmission” (92, 93). Further, peripheral (not only central) administration of kisspeptin successfully induced GnRH/gonadotropin release in mammals including rodents (53, 62, 64, 94), ruminants (46, 95), and primates (63, 96). This may be an advantage for therapeutic use of kisspeptin or its analogs in humans and domestic animals (97–99). Furthermore, GnRH neuronal cell bodies also seem to be an action site of ARC kisspeptin neurons because a retrograde tracing study revealed the projection of ARC kisspeptin neurons to the POA in mice (100) and confocal microscopy revealed contacts of kisspeptin fibers from the ARC population to GnRH neurons in ewes (101).

## Roles of Kisspeptin, Neurokinin B, and Dynorphin A in the Mechanism Controlling GnRH Pulse Generation

Theoretically, the GnRH pulse generator would consist of neurons that are connected to each other and show synchronized neuronal activity. The dense distribution of kisspeptin neuronal cell bodies and fibers in the whole ARC (54, 75) may be indicative of a neuronal connection between kisspeptin neurons. Such a dense kisspeptin-kisspeptin neuronal connection may be necessary to synchronize the release of kisspeptin to the GnRH neurons at the median eminence. Importantly, *Gpr54* is not found in ARC kisspeptin neurons in mice (66) and rats (68). Further, electrophysiology or MUA technology revealed that kisspeptin itself unlikely affects the activity of kisspeptin neurons in mice (102), rats (94), and goats (46), suggesting that other neuropeptide(s) may participate to synchronize the kisspeptin neuronal activity.

It is noteworthy that both neurokinin B and dynorphin A are co-localized in a majority of ARC kisspeptin neurons (therefore called KNDy neurons) in several mammalian species including rodents (7–11), ruminants (12–14), and primates (15, 16). Neurokinin B is one of the tachykinin family peptides, that are characterized by the presence of the common C-terminal amino acid sequence Phe-X-Gly-Leu-Met (or Leu)-amide (103) (**Figure 1B**). Among the tachykinin families, substance P, neurokinin A (both of which are cleaved from the same prepropeptide coded by *TAC1* gene in humans), and neurokinin B (which is cleaved from separate prepropeptide coded by *TAC3* gene) have been well-investigated, and the amino acid sequences of these peptides are identical in all mammalian species examined to date (103). There are three types of GPCRs for the tachykinins, denoted as NK1, NK2, and NK3 receptors. These receptors are recognized with moderate selectivity by endogenous tachykinins. Neurokinin B was reported to preferentially bind to the NK3 receptor, a Gq-coupled stimulatory GPCR (103). Neurokinin B previously attracted attention as a mediator for the hot flushes in postmenopausal women, who show ovarian steroid hyposecretion and gonadotropin hypersecretion (104). Importantly, inactivating mutations of the *TAC3* or *TACR3* (encoding the NK3 receptor) gene lead to hypogonadotropic hypogonadism in humans (105, 106), suggesting the importance of neurokinin B-NK3R signaling in human reproduction.

Dynorphin A is a family of endogenous opioid peptides characterized by the presence of the common N-terminal amino acid sequence Tyr-Gly-Gly-Phe-Leu (or Met) (107) (**Figure 1C**). There are three major endogenous opioids, such as β-endorphin, enkephalin, and dynorphin A, which are mainly cleaved from the separate prepropeptides encoded by *POMC*, *PENK*, and *PDYN*, respectively. Major corresponding receptors for β-endorphin, enkephalin, and dynorphin A are suggested to be μ-opioid, δ-opioid, and κ-opioid receptors, respectively, and the receptors are known as Gi-coupled inhibitory GPCRs (107). Dynorphin A was previously reported to be involved in negative feedback action of progesterone on GnRH pulse generation in ewes (108, 109).

Goodman and colleagues first found that neurokinin B and dynorphin A are largely co-localized in a single population of ARC neurons in ewes by immunohistochemistry for these peptides (110), and then uncovered that kisspeptin is also expressed in the same neuronal population (12). The co-localization of neurokinin B and dynorphin A in the ARC kisspeptin neurons were validated in several mammalian species such as goats (13), heifers (14), rats (9, 10), mice (7, 8, 11), and rhesus monkeys (15, 16) as summarized in **Table 1**. Co-localization of kisspeptin and neurokinin B was also found in humans (111–114) (**Table 2**), whereas few dynorphin A immunoreactivity was detected in the ARC kisspeptin/neurokinin B neurons in humans (112). Furthermore, NK3 receptors were found in a majority of rodent and ovine ARC KNDy neurons (7, 8, 11, 115, 116) and κ-opioid receptors were found in a majority of rat and ovine KNDy neurons and in a part of mouse KNDy neurons (7, 8, 11, 115, 117, 118) (**Table 3**). These findings suggest that the ARC KNDy neurons communicate with each other by neurokinin B-NK3 receptor signaling and dynorphin A-κ-opioid receptor signaling in an autocrine/paracrine manner. The species and sex differences in terms of the co-expressing rates of KNDy peptides and their receptors may imply the redundancy of KNDy neurons to maintain reproductive function in mammals as discussed later.

**Table 1:** Co-expression % of neurokinin B or dynorphin A in the arcuate kisspeptin neurons in ruminants, rodents, and rhesus monkeys.

Species	Sexes and treatments	NKB/Kp	Dyn/Kp	Methods	Reference no. and authors
Sheep	Female (OVX+E2)	–	94%	IHC	(12), Goodman et al., 2007
	Female (Ovary intact)^1^	80.4%	–	IHC	(12), Goodman et al., 2007
Goats	Female (OVX)	99.5%	78.0%	IHC	(13), Wakabayashi et al., 2010
Cattle	Female (Ovary intact)^2^	almost all^3^	>half^3^	IHC	(14), Hassaneen et al., 2016
Rats	Female (OVX)	97%	–	IHC	(9), True et al., 2011
	Female (OVX+E2)	majority^4^	majority^4^	IHC	(10), Murakawa et al., 2016
Mice	Female (OVX/OVX+E2)	90%	92%	ISH	(7), Navarro et al., 2009
	Male (Cast/Cast+T)	94%	86%	ISH	(8), Navarro et al., 2011
	Female (OVX)^5^	100%	100%	Pooled cell PCR	(11), Ikegami et al., 2017
Rhesus monkeys	Male (Cast)	40-60%	–	IHC	(15), Ramaswamy et al., 2010
	Female (OVX)	–	7.3%	IHC	(16), True et al., 2017

Cast, castration; Dyn, dynorphin A; E2, estradiol-17β; Kp, kisspeptin; NKB, neurokinin B; OVX, ovariectomy; T, testosterone.

^1^Data were collected from ewes at the luteal, follicular, and estrous stages.

^2^Data were collected from heifers at the luteal and follicular stages.

^3^Data were not shown as percentage.

^4^Data were not shown as percentage.

^5^Data were collected from Kiss1-GFP transgenic mice.

**Table 2:** Co-expression % of kisspeptin and neurokinin B in the arcuate nucleus of humans.

Species	Sexes	Ages	NKB/Kp	Kp/NKB	Methods	Reference no. and authors
Humans	Female	27-74 years old	77.0%	95%	IHC	(111), Hrabovszky et al., 2010
	Male	Young (21-37 years old)	75.2%	32.9%	IHC	(112), Hrabovszky et al., 2012
	Male	Young (21-49 years old)	72.7%	35.8%	IHC	(113), Molnar et al., 2012
	Male	Aged (50-78 years old)	77.9%	68.1%	IHC	(113), Molnar et al., 2012
	Female	Postmenopausal (64-90 years old)	78.4%	66.5%	IHC	(114), Skrapits et al., 2014

**Table 3:** Co-expression % of NK3 receptors or κ-opioid receptors in the arcuate kisspeptin/neurokinin B neurons.

Species	Sexes and treatments	NK3R	KOR	Targets	Methods	Reference no. and authors
Sheep	Female (Ovary intact)^1^	64%	–	NKB	IHC	(116), Amstalden, et al., 2010
	Female (Ovary intact)^1^	–	97.8%	Kp	IHC	(118), Weems et al., 2016
	Female (Ovary intact)^1^	–	93.5%	NKB	IHC	(118), Weems et al., 2016
Rats	Female (OVX+E2)	–	62%	Kp	ISH	(117), Tsuchida et al., 2020
Mice	Female (OVX/OVX+E2)	96%	20%	Kp	ISH	(7), Navarro et al., 2009
	Male (Cast/Cast+T)	76%	6%	Kp	ISH	(8), Navarro et al., 2011
	Male (Testis intact)^2^	35%	41%	NKB	Single-cell PCR	(115), Ruka et al., 2013
	Male (Cast)^2^	86%	19%	NKB	Single-cell PCR	(115), Ruka et al., 2013
	Female (OVX)^3^	83%	33%	Kp	Pooled cell PCR	(11), Ikegami et al., 2017

Cast, castration; E2, estradiol-17β; Kp, kisspeptin; KOR, κ-opioid receptors; NK3R, NK3 receptors; NKB, neurokinin B; OVX, ovariectomy; T, testosterone.

^1^Data were collected from ewes at the luteal stage.

^2^Data were collected from Tac2-GFP transgenic mice.

^3^Data were collected from Kiss1-GFP transgenic mice.

**Figure 2:**
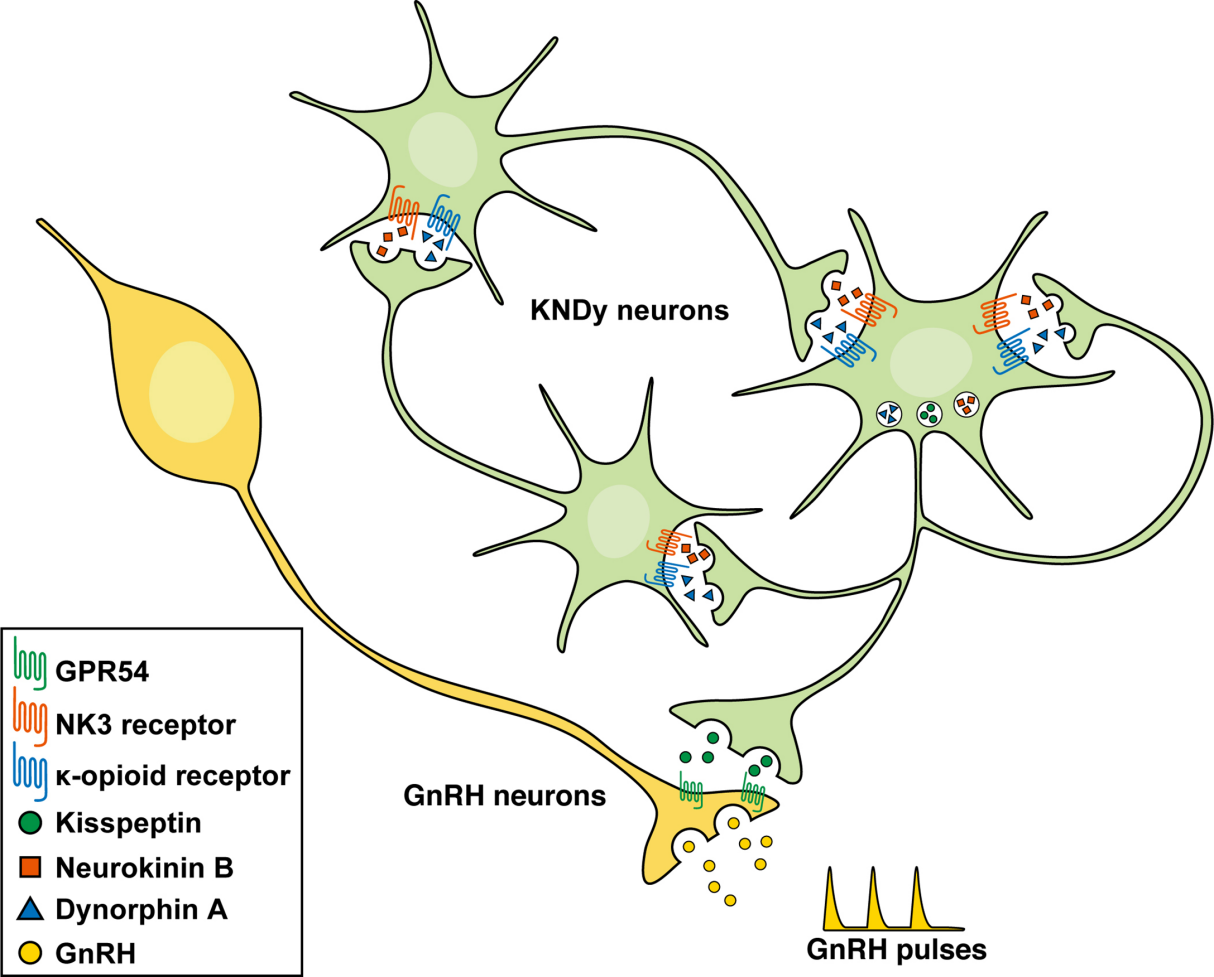
Schematic illustration of the hypothetical mechanism controlling gonadotropin-releasing hormone (GnRH) pulse generation in mammals. Neurokinin B initiates and/or accelerates synchronized KNDy neuronal activity *via* stimulatory Gq-coupled NK3 receptors to release kisspeptin that stimulates GnRH release *via* stimulatory Gq-coupled GPR54 expressed in GnRH neurons. Dynorphin A released from KNDy neurons then terminates KNDy neuronal activity *via* inhibitory Gi-coupled κ-opioid receptors.

**Figure 2** shows a schematic illustration of the hypothesis explaining the mechanism controlling GnRH pulse generation. The current most plausible interpretation is that neurokinin B initiates and/or accelerates synchronized KNDy neuronal activity *via* stimulatory Gq-coupled NK3 receptors to release kisspeptin that stimulates GnRH release *via* stimulatory Gq-coupled GPR54 expressed in GnRH neurons, and that dynorphin A released from KNDy neurons then terminates KNDy neuronal activity *via* inhibitory Gi-coupled κ-opioid receptors. Indeed, a central administration of neurokinin B facilitated the frequency of the MUA volley, which is corresponding the GnRH pulse generator activity, and the frequency was lowered by a central administration of dynorphin A and increased by nor-binaltorphimine (nor-BNI), a κ-opioid receptor antagonist, in female goats (13). Peripheral administrations of PF-4455242, another κ-opioid receptor antagonist, facilitated and SB223412, an NK3 receptor antagonist, suppressed LH pulses in female goats (119, 120). Similarly, a central administration of neurokinin B or nor-BNI facilitated and SB222200, another NK3 receptor antagonist, suppressed LH pulses in ewes (121). In addition, neurokinin B and senktide, an NK3 receptor agonist, increased the firing frequency of a majority of male mouse KNDy neurons (8, 102) and dynorphin A and U50-488, a κ-opioid receptor agonist, decreased the firing frequency of all the KNDy neurons tested (102). Taken together, it is most likely that neurokinin B serves as a stimulatory signal for ARC KNDy neurons and dynorphin A serves as an inhibitory signal for the neurons, leading to the synchronized pulsatile pattern of the KNDy neuronal activity to generate GnRH pulse.

## Direct Evidence That KNDy Neurons as the GnRH Pulse Generator

As we mentioned above, circumstantial evidence accumulated in the last 15 years suggests that kisspeptin, neurokinin B, and dynorphin A in KNDy neurons play key roles in controlling pulsatile GnRH release in female mammals including rodents (58, 89, 91) and ruminants (13, 46, 121, 122). However, no direct evidence proving the role of KNDy neurons as the GnRH pulse generator had been provided yet because gene-modified mice and rats lack *Kiss1* expression in both two populations of hypothalamic kisspeptin neurons as well as extra-hypothalamic and peripheral kisspeptin-producing cells. For example, we previously generated global *Kiss1* knockout rats to demonstrate the indispensable role of kisspeptin in both pulsatile and surge-mode GnRH/gonadotropin release (58). The global *Kiss1* knockout rats clearly reproduced the hypogonadal phenotypes of human and mouse models carrying *KISS1*/*Kiss1* or *GPR54*/*Gpr54* mutations such as pubertal failure and atrophic gonads in both sexes. Importantly, the *Kiss1* knockout rats exhibited a complete suppression of pulsatile LH release even after gonadectomy in both sexes, suggesting that kisspeptin neurons serve as the GnRH pulse generation in both sexes. In addition, global *Kiss1* knockout female rats exhibited no LH surge when animals were treated with preovulatory levels of estradiol-17β.

To prove that KNDy neurons serve as the GnRH pulse generator, we rescued KNDy neurons by infecting viral vectors expressing *Kiss1* mRNA targeted into the ARC *Tac3*-expressing neurons in global *Kiss1* knockout female rats (123). Pulsatile LH release was recovered in KNDy-rescued rats in which 20-50% ARC *Tac3*-expressing neurons exhibited *Kiss1* expression. The profiles of LH pulses are largely dependent on the rescue rates of KNDy neurons, indicating that the rescue of KNDy neurons, but not *Kiss1* transfection outside of ARC *Tac3*-expressing neurons, could recover LH pulses in global *Kiss1* knockout female rats. Further, rescuing KNDy neurons could recover folliculogenesis, but not ovulation, suggesting that KNDy neurons are largely responsible for GnRH/LH pulse generation but not surge generation. To confirm the notion obtained by the KNDy rescue experiment, we evaluated the effect of conditional ARC *Kiss1* knockout on GnRH pulse generation in newly generated *Kiss1*-floxed rats. By using the Cre-loxP system, we engineered conditional ARC *Kiss1* knockout rats (123). Pulsatile LH release was completely suppressed in conditional ARC *Kiss1* knockout female rats in which >90% *Kiss1*-expressing cells disappeared in the ARC.

The finding that 20% of KNDy neurons are enough to maintain GnRH pulses and folliculogenesis in the rat suggests the functional redundancy of the KNDy neuronal population. The notion of redundancy is also supported by a previous study showing that gene-modified mice bearing <5% *Kiss1* expression still exhibited puberty and fertility in both sexes (124). To date, little is known the functional redundancy of the KNDy neurons in non-rodent mammalian species.

It should be noted that the previous immunohistochemical studies showed co-localization of NK3 receptor in a number of GnRH fibers in rats (125) and κ-opioid receptor in a majority of GnRH cell bodies in rats and ewes (118, 126). These findings imply that neurokinin B and dynorphin A may also directly acts on GnRH neurons to control GnRH release. Nevertheless, it is unlikely that neurokinin B and/or dynorphin A derived from KNDy neurons directly act on GnRH neurons to participate in GnRH pulse generation. This is because plasma LH/FSH levels were undetectable in global *Kiss1* knockout rats (58), in which *Tac3* and *Pdyn* gene were abundantly expressed in the ARC as shown in wild-type female rats. Further, *Kiss1* rescue into the ARC *Tac3*-expressing cells but not out of the *Tac3*-expressing cells rescued LH pulses in the global *Kiss1* knockout female rats (123). These findings indicate that ARC neurokinin B/dynorphin A neurons without *Kiss1* could not drive GnRH/LH pulse generation. In this context, non-KNDy neurokinin B or dynorphin A neurons may directly project GnRH neurons and control/modulate GnRH and consequent gonadotropin release. Supportedly, our recent study suggested that the dynorphin neurons derived from the hypothalamic paraventricular nucleus mediate glucoprivic suppression of LH pulses (117).

## Conclusions, Unanswered Questions, and Future Aspects

Kisspeptin discovery in the early 2000s and its subsequent studies have provided a breakthrough in our understanding of the brain mechanism underlying reproduction in mammals along with the rediscovery of the critical roles of neurokinin B, a tachykinin, and dynorphin A, an endogenous opioid peptide. We now postulate that KNDy neurons act as an intrinsic source of the GnRH pulse generator, in which neurokinin B serves as a stimulatory signal, dynorphin A serves as an inhibitory signal, and kisspeptin serve as an output signal of KNDy neurons that drive GnRH release from the GnRH neurons. There are, however, still some unanswered questions. For example, there are reportedly species differences in neurokinin B signaling in KNDy neurons: The NK3 receptor antagonist SB223412 potently inhibited gonadotropin and testosterone release in male dogs (127), on the other hand, CS-003, a triple tachykinin receptor antagonist, was needed to inhibit LH secretion in male and female rats (128). Similarly, electrophysiology revealed that three tachykinin receptors were needed to be antagonized to prevent the stimulatory action of NKB on male mouse KNDy neurons *in vitro* (102). There are also species differences in terms of the co-expressing rates of dynorphin A and κ-opioid receptors in KNDy neurons as described above. Further, the sex differences in the number of ARC kisspeptin/neurokinin B neurons were reported in humans: males have fewer kisspeptin- and neurokinin B-positive cells in the ARC than females (111, 129). The sex differences could be caused by feedback from different endogenous steroids in gonad-intact human subjects as reviewed elsewhere (122, 130). Indeed, previous studies showed the increases in *KISS1* and *TAC3* expression in the ARC of postmenopausal women compared to premenopausal women (84, 104). Further studies are warranted on how neurokinin B and dynorphin A orchestrate the synchronized activity of KNDy neurons and GnRH pulses in mammals and the significance behind the species and sex differences to contribute to future therapeutic approaches in both humans and domestic animals suffering from reproductive disorders.

## Author Contributions

YU and HirT collected the information and wrote the manuscript. NI designed the pictures and critically revised the manuscript. MN and HitT collected the information and critically revised the manuscript. All authors contributed to the article and approved the submitted version.

## Funding

The present study was supported in part by JSPS KAKENHI Grant Numbers 18H03973, 21K19186, and 21H05031 (to HirT), 19H03103 (to NI), 20H03127 (to YU).

## Conflict of Interest

The authors declare that the research was conducted in the absence of any commercial or financial relationships that could be construed as a potential conflict of interest.

## Publisher’s Note

All claims expressed in this article are solely those of the authors and do not necessarily represent those of their affiliated organizations, or those of the publisher, the editors and the reviewers. Any product that may be evaluated in this article, or claim that may be made by its manufacturer, is not guaranteed or endorsed by the publisher.
